# Deformation analysis of 3D tagged cardiac images using an optical flow method

**DOI:** 10.1186/1532-429X-12-19

**Published:** 2010-03-30

**Authors:** Chun Xu, James J Pilla, Gamaliel Isaac, Joseph H Gorman, Aaron S Blom, Robert C Gorman, Zhou Ling, Lawrence Dougherty

**Affiliations:** 1 Gorman Cardiovascular Research Group, Glenolden Research Laboratory, University of Pennsylvania, Glenolden, PA, 19036, USA; 2 Department of Radiology, 1 Silverstein, 3400 Spruce Street, Philadelphia, PA 19104, USA

## Abstract

**Background:**

This study proposes and validates a method of measuring 3D strain in myocardium using a 3D Cardiovascular Magnetic Resonance (CMR) tissue-tagging sequence and a 3D optical flow method (OFM).

**Methods:**

Initially, a 3D tag MR sequence was developed and the parameters of the sequence and 3D OFM were optimized using phantom images with simulated deformation. This method then was validated *in-vivo *and utilized to quantify normal sheep left ventricular functions.

**Results:**

Optimizing imaging and OFM parameters in the phantom study produced sub-pixel root-mean square error (RMS) between the estimated and known displacements in the x (RMS_x _= 0.62 pixels (0.43 mm)), y (RMSy = 0.64 pixels (0.45 mm)) and z (RMSz = 0.68 pixels (1 mm)) direction, respectively. *In-vivo *validation demonstrated excellent correlation between the displacement measured by manually tracking tag intersections and that generated by 3D OFM (R ≥ 0.98). Technique performance was maintained even with 20% Gaussian noise added to the phantom images. Furthermore, 3D tracking of 3D cardiac motions resulted in a 51% decrease in in-plane tracking error as compared to 2D tracking. The *in-vivo *function studies showed that maximum wall thickening was greatest in the lateral wall, and increased from both apex and base towards the mid-ventricular region. Regional deformation patterns are in agreement with previous studies on LV function.

**Conclusion:**

A novel method was developed to measure 3D LV wall deformation rapidly with high in-plane and through-plane resolution from one 3D cine acquisition.

## Background

Functional alterations in left ventricular (LV) myocardium have been demonstrated as important indicators of cardiac disease [1]. Accordingly, the ability to accurately quantify the motion and deformation of the intact heart is fundamental to understanding cardiac mechanics, improving diagnosis and developing treatments. Despite its importance, the conventional modalities to quantify the heart wall motion and function are limited to the heart wall surfaces, and are not able to properly account for the motion through the imaging plane. Thus, they are largely applied to assess global ventricular functions, such as the ejection fraction, or qualitative functions of the regional heart wall. To compute the complex myocardial deformation requires the visualization and tracking of material points in the left ventricular wall which previously has relied on implanted ultrasonic or radiopaque markers [2-4], which is limited by its invasive nature and poor resolution. The introduction of tissue-tagging by Zerhouni *et al. *[5] and Axel *et al. *[6] allowed for precise tracking of non-invasive material points within the heart wall. As a result, tissue tagging has become the gold standard to study multidimensional myocardial motion in both normal [7,8] and diseased hearts [9].

One of the major challenges associated with CMR tagging is quantitative analysis. Numerous methods have been proposed to reconstruct cardiac motion from tagged CMR images. Currently, most analysis is two dimensional (2D) [10,11], which has limited ability to precisely capture the complex radial, circumferential and longitudinal motion associated with normal and diseased hearts [12]. Among the proposed three dimensional (3D) analysis techniques is a deformable model which is fit to either the tag line material points extracted from the image data [13-16], or the image data set using non-rigid registration [17]. These approaches are based primarily on finite element [18,19], Cartesian B-spline [15,16], finite difference [14], and free-form deformations [17]. Pre-definition of a 3D LV deformable model is mandatory, which requires guide points and subsequent fitting of the model's deformation to the motion of the tagged points in two views [18,19], fitting of a 3D displacement field composed of analytic series [13], or user-defined myocardial contours [16].

The main challenges associated with previous 3D analysis methods include motion field reconstruction from a collection of sparse displacement measurements as well as time-consuming post-processing procedures. In addition, acquisition of tagged images for these methods currently consists of 2-3 orthogonal image sets acquired separately or the acquisition of multiple encoding directions. These long scan times result in errors due to mis-registration and physiological changes that decrease the accuracy of the measurements. While these available techniques have shown significant potential for qualitative assessment, an approach that allows direct measurement of 3D tissue displacements from 3D tagged images with greater accuracy and higher spatial resolution is essential to study normal cardiac mechanics and pathology in the clinical arena.

A method used for tagged CMR analysis which has received recent attention is the harmonic phase method (HARP) [20], which uses tag phase to estimate the underlying tissue displacements. For 3D analysis, HARP uses two orthogonal acquisitions that are combined during analysis to give regional strain estimates with less than optimal spatial resolution. HARP is also prone to error in regions where magnetic susceptibility is an issue, such as the blood-myocardium interface [21]. In addition, the spectral DC peak can interfere with the motion estimation thus decreasing accuracy [22]. Slice following with HARP has also been investigated as a true 3D tracking method but the spatial resolution is still limited to intersection points [23]. Slice following has also been applied to displacement encoding with stimulated echoes (DENSE) to track 3D cardiac motion but this approach is limited by reduced image signal to noise ratio and the time required to slice encode in three directions [24].

A 2D Optical Flow Method (OFM) has previously been used to estimate LV deformation from tagged CMR images [25]. This approach used Laplacian and Gaussian filtering combined with a hierarchical estimation technique to compute the motion that described the alignment of sequential images [26,27]. This method is particularly suited for analyzing tagged cardiac images due to the high-resolution and high-contrast tag lines that serve as landmarks for confident pixel identification and tracking. Two-dimensional OFM has been validated using a silicon gel phantom with simulated rotational motion [25], and demonstrated the ability to track each pixel in a defined region of interest (ROI) rapidly and with minimal user interface.

To overcome the limitations associated with conventional methods to evaluate global and regional cardiac functions, this study proposes a novel approach that combines 3D tagged CMR and 3D OFM to track in-plane and through-plane cardiac motion from one single cine acquisition. The method is optimized and validated on a simulation phantom (*ex-vivo*) and subsequently evaluated *in vivo *using an ovine model. The effect of noise on 3D tracking is investigated and the accuracy of 2D tracking of 3D displacement is examined.

## Methods

### 3D Tag Pulse Sequence Combined with 3D OFM

In the conventional approach to tagged-CMR, 2D tags are applied to the heart as an orthogonal grid in the plane of the image (*x*-*y*). With 2D tags alone, accurate analysis is limited to motion that is within the plane of the tags due to a lack of adequate landmarks with which to resolve the ambiguity in the third dimension. This ambiguity is in part due to the aperture problem, which shows that motion can only be detected which is normal to the tag lines. In order to estimate 3D motion, an additional image series would be acquired with a single tag plane applied sensitive to motion in the slice direction (*z*). These image series would then be combined using numerical methods to estimate 3D displacement. In the approach described here, a third set of tags is applied within a single acquisition sequence, which resolves the aperture problem and allows for the subsequent analysis with 3D OFM.

A pulse sequence was developed that used three SPAMM tag sets, each comprised of five RF pulses with a composite flip-angle of 130°. The SPAMM tags were applied at the detection of the cardiac trigger followed by multi-phase image acquisition using a 3D-gradient echo imaging sequence. Two tag sets created a grid orthogonal to the imaging plane, as in a 2D acquisition, while the third set created tags that were sensitive to through-plane motion. Although it would seem obvious to apply the third set orthogonal to the other tags, in practice this is not desirable. A tag is created by saturating the spins to create contrast. If the third set was orthogonal to the in-plane grid, and therefore parallel to the imaging plane, then entire slices would be saturated and all in-plane information from those regions would be lost. Instead, we investigated applying the third tag set at an oblique angle, which must be chosen carefully to balance sensitivity to through-plane motion without too much loss of signal from the saturated regions. Optimization of the tag angles is described in more detail below.

The OFM used in this study is a coarse-to-fine motion estimation technique that is performed in a multi-step procedure. It first estimates a global parametric transformation (translation and rotation) between two images and then, using the global transformation as an initialization, proceeds to an estimation of the local flow vector for each point between the two images. The tagged multi-phase images were sequentially pair-wise compared to compute the flow field and were then vector integrated to form the overall local displacement.

Image analysis was started by pre-processing the images using a 3D Laplacian filter to eliminate the local image intensity offset and to enhance the edges, making image alignment more sensitive to structure as opposed to overall intensity [25,26]. Within each estimate stage, the image was decimated multiple times to create a pyramid of coarse-to-fine resolution levels ("Laplacian pyramids"), producing a set of bandpass components. The 3D voxel velocity *u (r) *was then computed for each adjacent pair of images *(L) *by minimizing the sum of the square difference (SSD) between the pair (*Li (t), Li(t+1)*). The SSD at each voxel is given by:(1)

Where *L*_*i *_is the Laplacian pyramid image intensity; *i *is the pyramid level; *r *is the 3D spatial location; ***u****(r) *is voxel velocity.

The minimization of Equation (1) with respect to ***u ***is an iterative process. For each iteration, the incremental change in velocity is computed using the Gauss-Newton optimization technique. The error due to incremental change in velocity for the kth iteration is given by:(2)

For global estimates of motion, the region of interest (ROI) is the entire image, whereas for local estimates, the inspection window is a 3 × 3 region around each pixel. Spatial gradients are computed from forward and backward differences (+/- half the window width) and then averaged. The incremental estimate ∂***u ***is computed and added to the current estimate ***u***_*k *_to obtain a new estimate ***u***_*k*+1_. This process is repeated for a fixed number of iterations or until there is convergence, which is tested using the SSD. When there is no reduction in the SSD, the process moves to the next pyramid level. This is performed sequentially, in coarse to fine order, through each level of the pyramid and provides an initial estimate for the succeeding level [26,27]. The optimal number of pyramid levels and OFM iterations were chosen from the simulation described below.

### Optimization and Validation

All animals used in this study receive care in compliance with the *Guide for Care and Use of Laboratory Animals *published by National Institutes of Health (NIH publication 86-23, revised 1996). The protocol was approved by the Institutional Animal Care and Use Committee of the institution.

Optimisation and validation of the 3D tag imaging parameters and 3D OFM technique was performed using a synthetic 3D tagged image sets generated from ex vivo heart images. Initially, a 3D-image volume was acquired of an ovine heart following the termination of an unrelated experiment. The sheep was euthanized and the heart was extirpated and fixed in formalin. The heart was then imaged in a 1.5T Siemens Sonata scanner (Siemens Medical Solutions, Malvern, PA, USA) using a segmented FGRE sequence with scan parameters: TR = 7 ms, TE = 2.2 ms, FOV 180 × 180 cm, matrix 256 × 256, BW 388 Hz/pixel, slice thickness = 1.5 mm, FA = 15°. Synthetic tags were superimposed on the static images employing a method similar to that used by other researchers [28,29]. The method involved creating a uniform intensity image set with the same dimensions as that of the *ex-vivo *heart. Subsequently, independent grids composed of horizontal, vertical, and oblique tag lines were defined by setting intensity of the lines to saturation (zero). The resulting 3D tagged phantom heart images were generated by using matrix multiplication to combine the ex-vivo heart images with the synthetic tags.

**Figure 1:**
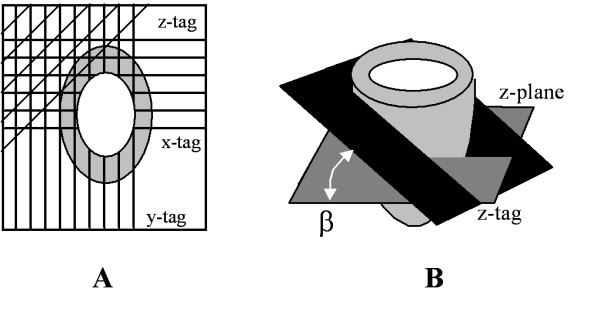
**Representative LV with 3D tag planes superimposed**. The third tag plane is oriented 45° relative to the in-plane tags (***x***-tag, ***y***-tag) (**A**) and the angle relative to the ***z***-axis is represented by β (**B**). The three tag planes create dark stripes on the LV wall, which serve as landmarks for 3D OFM tracking.

Tag plane orientation was adjusted by keeping the in-plane orthogonal tags fixed in space and the angle between these tags and the third tag plane constant at 45°. The angle between the ***z***-axis and the third tag plane (β) was varied from 0° (parallel to **z**-axis) to 90° (perpendicular to ***z***-axis) in 15° increments (Figure 1). Left ventricular 3D displacement maps from an independent normal ovine heart was extracted, scaled and used to warp the synthetic image set to simulate systolic LV motion. *T*_1 _dependent tag fading through the cardiac cycle was reproduced using *SI *= *M*_0_(1 - *e*^-*t*/*T*1^) with *T*_1 _= 900 ms. Phase-to-phase displacement of the phantom was estimated using 3D OFM [25,30], which is discussed previously.

Tag parameter simulations were performed using multiple combinations of width, spacing and angle with in-plane tag spacing chosen to be a minimum of two tags across the LV wall. Through-plane and in-plane tags parameters were adjusted independently. OFM parameters investigated in this study included the number of pyramid levels, the number of iterations at each level, and the filtering method (Laplacian/Gaussian). The OFM as well as the 3D tag parameters were chosen to minimize the error between the prescribed and estimated displacement. Tracking error was defined as the root-mean-square (RMS) over all myocardial pixels.

*In-vivo *validation was performed using a tagged 3D dataset acquired from a normal sheep using the identical imaging protocol described later. Three-dimensional systolic LV motion was measured using 3D OFM and subsequently applied to the tagged image set acquired at ED. Displacement was validated by manually comparing the intersection coordinates of the three tag planes from the actual ES images to those obtained from the ED images warped by the OFM measured displacement. Linear regression analysis between the warped and actual ES points was performed using a total of 66 3D tag intersection points evenly distributed over the entire LV [31].

Through-plane displacement introduces error in the 2D tag strain measurements due to tissue rotation being projected into the in-plane displacement [24]. Quantification of the error incorporated into the 2D displacement due to 3D motion was measured using 3D OFM. Two-dimensional OFM has previously been optimized, validated and was shown to have less than 5% error for 2D motion tracking [25]. To demonstrate the advantages in accuracy of 3D over 2D OFM in measuring cardiac displacement, 2D OFM was used to extract the in-plane projection of 3D motion using the same synthetic tagged image and the results were compared to 3D in-plane tracking.

Noise can adversely affect the accuracy of the computed displacement due to the random variation of pixel intensity and location. Since strains are calculated from the derivative of displacement, they are particularly sensitive to image noise. Therefore, to test the susceptibility of the 3D OFM algorithm to noise, zero mean Gaussian noise (5-20%) was applied to the baseline synthetic tagged image dataset and the RMS was calculated as described above.

### In Vivo Study

Five normal Dorset sheep were scanned using the optimized OFM and tag parameters gleaned from the phantom experiment. Animals were induced with thiopental sodium (10 to 15 mg/kg IV) and intubated. Anesthesia was maintained with isofluorane (1.5% to 2%) and oxygen. A Millar pressure catheter was placed in the LV cavity to monitor the animal and to serve as cardiac gating. Imaging was performed on a 3T Siemens TIM Trio scanner (Siemens Medical Solutions, Malvern, PA, USA) using a 3D fast gradient echo sequence with a 3D tag preparatory pulse. Respiratory and LV pressure cardiac gating were used to minimize respiratory and cardiac motion. Anterior and posterior phased array flex coils were placed on the animal and imaging was performed using the following parameters: TR/TE/FA = 5.6 ms/2.53 ms/15^0^, Averages = 3, views per segment = 4, slice thickness = 3 mm, raw data matrix 256 × 128 interpolated to 256 × 256, rectangular field of view 220 mm × 220 mm, pixel size 0.86 × 0.86 mm, tag spacing = 7 mm, BW 388 Hz/pixel, 12-22 slices were acquired depending on heart size, heart rate 100 ± 8 bpm, average scan time 25 minutes with gating.

### Strain Analysis

Using the optimized 3D OFM, the pixels of each slice were tracked through systole with sub-pixel resolution. LV endocardial and epicardial contours were manually drawn on every other slice at one mid-systolic phase and then automatically interpolated in both temporal and spatial directions to obtain contours at each phase and location. Subsequently, displacements of each pixel were estimated using 3D OFM, which has been integrated into ImageJ http://rsb.info.nih.gov/ij/index.html.

**Figure 2:**
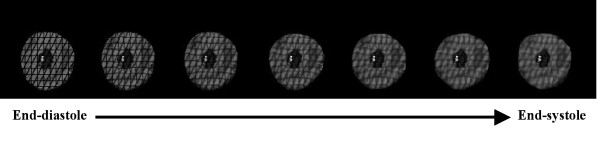
**Mid-ventricular slice from the synthetic left ventricular volume deformed from end-diastole (left) to end-systole (right)**. Three tag planes are shown in the image: two through-plane tag planes and one oblique tag plane. Blood in the LV cavity and tags appear black while the myocardium appears bright. Tag fading through the cardiac cycle due to *T*_1 _relaxation is evident by the decrease in tag CNR at end-systole.

To quantify tissue deformation, Lagrangian strain was derived by analytically differentiating the cardiac displacement between the initial state of end-diastole (ED) and the deformed state of end-systole (ES) (Figure 2). The deformation gradient tensor, ***X***, during time *0 *to *t*, was defined as:(3)

The Green-Lagrange strain tensor is a method suitable for large deformation estimation [32] and was computed by:(4)

where ***I ***denotes the identity matrix. Additionally, to assess LV local deformation independent of a coordinate system, the corresponding eigenvalue problem was solved to evaluate the principal strain magnitudes (***ε***_1 _and ***ε***_3_), and the eigenvector (α) of the first (or maximum) principal strain for each element. A positive strain value indicates lengthening or thickening, while a negative strain value indicates shortening. α is defined as the absolute value of the angle between the direction of the greatest stretch and the line connecting the centre of the endocardial contour with the pixel studied. Maximum principal strain (***ε***_1_) is interpreted as radial wall thickening while; minimal principal strain (***ε***_3_) is a combination of circumferential-longitudinal shortening.

### Regional Functional Analysis

For comparison between animals, regions were defined in which the average strain was calculated. The anterior right ventricle (RV) insertion point was identified and used as a reference point from which the LV was divided into twelve circumferentially equal sectors. Anatomical regions for the sectors were defined as: anteroseptal (AS), posteroseptal (PS), posterior (PST), posterolateral (PL), anterolateral (AL) and anterior (ANT). Longitudinal segments were defined by sectioning the long-axis into three equal segments (apical, mid-ventricular and basal) between the basal and apical.

## Results

### Phantom and In-vivo Validation

Figure 2 shows the time course of a representative mid-ventricular slice of the synthetic tagged image set from ED to ES. The entire image stack was analyzed as described below with the exception of the two most apical slices due to difficulty in segmentation.

The effects of 3^rd ^tag plane angles on RMS values are shown in Table 1. The measured in-plane (*x, y*) displacement exhibits lower RMS errors compared to the through-plane (*z*) displacement. The optimal tag angle for through-plane motion occurred when it was parallel to the imaging plane (β = 0°). However, this configuration reduced the overall spatial resolution due to saturation of slices from the application of the tags. The oblique tag angle that achieved the lowest error in the ***z***-direction while preserving spatial resolution was 30°. The optimal tag thickness and spacing were determined to be 2 and 6 pixels respectively. The lowest error was achieved using OFM parameters: 3 coarse-to-fine levels; 8 iterations at each level; Gaussian filtering for the global estimations; 1 level; 8 iterations and Laplacian filtering in the local stage.

**Table 1:** RMS tracking errors (in pixels) of estimated displacements compared with known flow fields measured in three Cartesian directions (*x*, *y *and *z*).

β Angle	RMS_x_	RMS_y_	RMS_z_
**0**	0.627	0.634	0.687
**15**	0.626	0.640	0.705
**30**	0.626	0.645	0.689
**45**	0.629	0.646	0.705
**60**	0.649	0.677	0.832
**75**	0.631	0.652	0.776
**90**	0.638	0.654	0.945

Orientation of the third oblique tag plane is controlled by adjusting the angle between the *z*-axis and the tagging plane (β).

**Figure 3:**
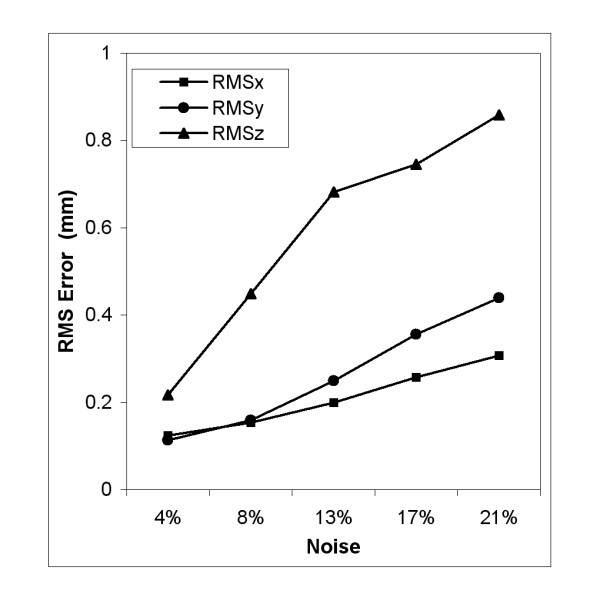
**RMS error of the displacement derived from the optimized synthetic phantom with added noise compared to noise-free baseline model**. Noise has a greater impact in the longitudinal through-plane (***z***-) direction than in the ***x***- and ***y***- in-plane directions.

Gaussian noise was applied to the baseline synthetic tagged images and RMS errors (relative to the noiseless baseline estimation) of systolic flow in ***x***, ***y ***and ***z ***direction were computed. Figure 3 shows the sensitivity of displacement estimation to spatial noise using 3D OFM. RMS error of in-plane displacement was below 0.5 mm for noise levels less than 20% of maximum image intensity. Through-plane motion estimation was more sensitive to added noise than in-plane measures and showed greater error overall.

The integrated left ventricular systolic displacement of synthetic tagged images was estimated using both 2D and 3D OFM. During systole, displacements computed by both OFM schemes demonstrate significant motion directed inward towards the LV cavity with reduced displacement observed close to the septum. However, 3D OFM yields a more uniform motion pattern and clockwise rotation near the base and counter-clock rotation at the apex. 3D OFM demonstrates the ability of detecting through plane motion (i.e. basal-apex direction), as well as more accurate measurements of in-plane motion resulting in a 51% decrease in the RMS compared to the 2D method. The RMS of 3D estimates in each direction was 0.43, 0.45, and 1 (mm) compared to 0.9, 0.92, and N/A (pixels) for the 2D method.

An in vivo validation was performed using the manually tracked intersection points from the actual ES displacement and the displacement measured by OFM. The values obtain from both methods were strongly correlated (R ≥ 0.98) indicating agreement between the actual and measured displacements.

### In vivo

**Figure 4:**
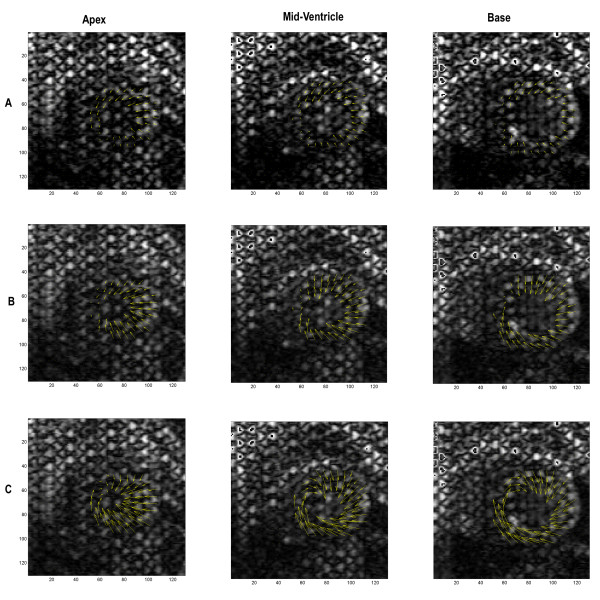
**In-plane displacement from the LV of a representative animal for three systolic time-points: early-systole (A), mid-systole (B), and end-systole (C) at 3 locations: apex, mid-ventricle and base**. Anatomic marks (Sep = septum, Post = posterior, Lat = lateral, Ant = anterior) were marked for orientation. At early-systole, the entire LV demonstrates twisting and counter clockwise rotation close to the septum. At mid-systole, the apex and mid-ventricular regions show greater motion towards the cavity, with clock-wise posterolateral wall rotation in the basal section (viewed from apex to base). Conversely, counter clock-wise rotation can be observed at the anteroseptal wall in the apical section, producing a twisting between apex and base. At end-systole, the apical region demonstrates significant inward motion, accompanied by clock-wise rotation close to the septal wall. Basal and mid-ventricular segments are dominated by counter clock-wise rotation, with inward motion on the anterolateral wall.

The detailed 3D displacement patterns derived from the heart of a representative animal are illustrated in Figure 4, which shows the in-plane displacement patterns at three locations: basal, mid-ventricular and apical regions; and three systolic time points: early systole (4A), mid-systole (4B) and end-systole (4C). In general, displacement increases throughout systole with the basal and lateral free wall having the greatest myocardial displacement in the longitudinal and circumferential directions. At the start of systole, viewing from apex to base, the entire LV exhibits counter clock-wise rotation close to the septal wall. Simultaneously, the heart begins to shorten in the apex-base direction and to contract at the lateral free wall. At mid-systole, displacement towards the cavity is the dominant motion particularly in the apical and mid-ventricular regions, while clock-wise rotation can be observed at the posteroseptal wall in the basal region. Conversely, counter clock-wise rotation at the anteroseptal wall, accompanied with contraction at the posteroseptal wall can be observed in the apical segments, thus producing an opposite rotation between the base and apex creating a "wringing" effect. At the end of systole, the lateral and posterolateral apical regions demonstrate significant inward motion, while the remaining apical segments exhibit clock-wise rotation. Basal and mid-ventricular segments are dominated by counter clock-wise rotation with inward displacement at the anterolateral wall.

**Figure 5:**
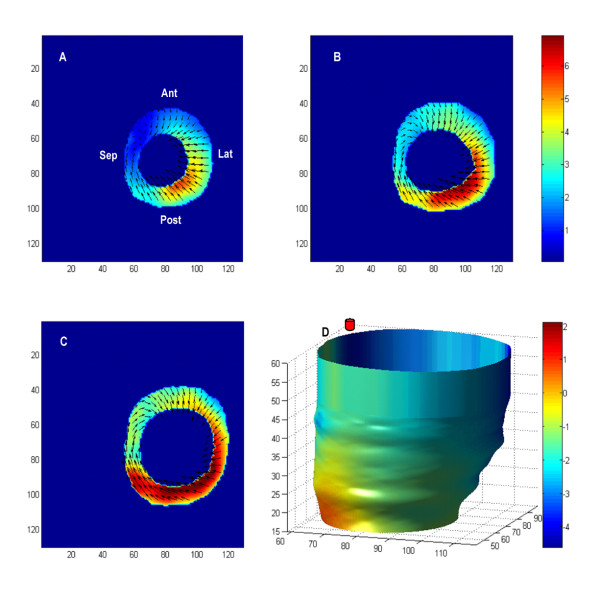
**Integrated systolic LV displacement of a representative animal**. Anatomic labels (Sep = septum, Post = posterior, Lat = lateral, Ant = anterior) have been added for orientation. Three short axis planes, apex (**A**), mid-ventricle (**B**), and base (**C**), were color-coded for 3D displacement magnitude (mm), and overlaid on the projection of the 3D displacement vectors. The length and direction of the arrows represent the magnitude and direction of the in-plane component of displacement respectively. LV mid-wall surface was reconstructed and color-coded with through-plane motion (mm) (**D**). The position of the septum is marked for reference (red circle).

Figure 5(A-C) shows systolic displacement integrated from ED to ES for the same three short-axis slices represented in Figure 4. Direction of the in-plane component of displacement is depicted as vectors and the magnitude as a colour-coded overlay. The plots reveal a non-uniform distribution with the greatest displacement occurring close to the basal regions. On each image, less radial displacement is observed close to the anteroseptal wall, specifically at the anterior RV insertion point, while maximum radial motion occurs close to posterolateral wall. Additionally, the endocardium shows greater motion than the epicardial wall. The vector plots also demonstrate more displacement on the anterior and anterolateral walls, with increased counter-clockwise rotation on the posterior and posterolateral walls. Longitudinal shortening during systole is shown in Figure 5D, which depicts a reconstructed LV mid-wall surface with colour-coded through-plane motion superimposed. The through-plane motion indicates that the apex is relatively stationary (-1.3 ± 0.8 mm) while the base exhibits more significant motion towards the apex (-3.5 ± 1.2 mm). The gradient of longitudinal displacement, however, is greater between mid-ventricular and apical sections, indicating that the basal region shows more rigid motion (translation) and less apex-base shortening (deformation).

**Figure 6:**
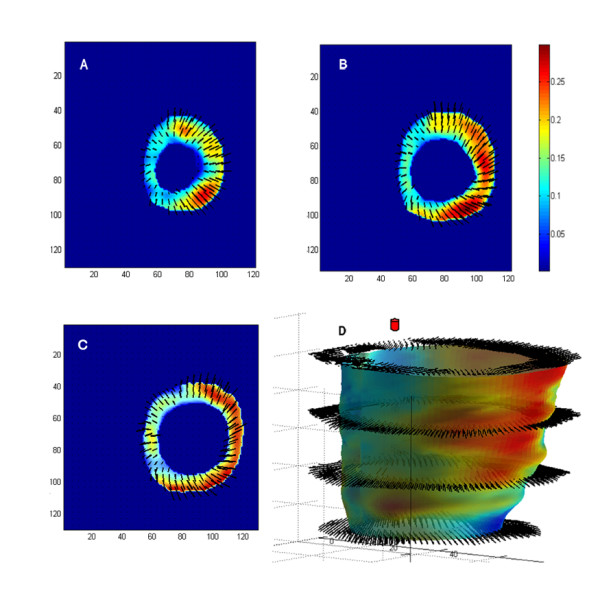
**LV end-systolic maximum principal strain (*ε*_1_) at the same longitudinal levels as depicted in Figure 5 (A-C)**. The color and length of the overlaid line segments represent the magnitude of ***ε***_1_, while the vector indicates the direction. The mid-wall surface was reconstructed and color-coded by eigenvalues ***ε***_1_, with eigenvectors superimposed at four longitudinal levels (**D**).

Figure 6(A-C) illustrates the maximum principal strain (***ε***_1_) on the three representative short axis images described above. The colour of the plot and the length of the overlaid line segments correspond to the magnitude of the strain while the direction is indicated by the vector. Myocardial thickening is generally aligned with the radial direction (20 ± 8 degrees) and the basal segment demonstrates more significant wall thickening than the apical and mid-ventricular regions with the exception of the basal-anterior region. The images show that the septum has the minimum ***ε***_1 _whereas the lateral wall has the maximum. Figure 6D shows a mid-wall surface colour-coded by with ***ε***_1_, with eigenvector direction at four longitudinal levels superimposed. It is evident that ***ε***_1 _is directed towards the centre of the LV indicating in-plane radial thickening.

**Figure 7:**
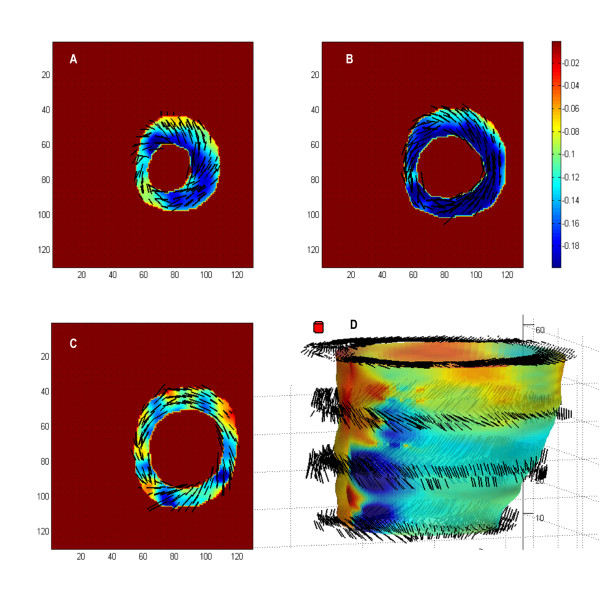
**LV end-systolic minimum principal strain (*ε*_3_) at the same longitudinal levels as depicted in Figure 5 (A-C)**. The color and length of the overlaid line segments represent the magnitude of ***ε***_3_, while the vector indicates the direction. The mid-wall surface was reconstructed and color-coded by eigenvalues ***ε***_3_, with eigenvectors superimposed at four longitudinal levels (D).

Minimum principal strains (***ε***_3_), which are analogous to myocardial shortening that is approximately parallel to endocardium, are shown in Figure 7. Panel C and D indicate that ***ε***_3 _at base has lower magnitude compared with other regions and primarily circumferential shortening, confirming that the base has more rigid longitudinal motion than deformation (***ε***_3 _= -0.12). In contrast, myocardial shortening in apical and mid-ventricular regions is due to the combination of circumferential and longitudinal shortening, with greatest magnitude of shortening occurs in the mid-ventricular segment (***ε***_3 _= -0.2).

**Figure 8:**
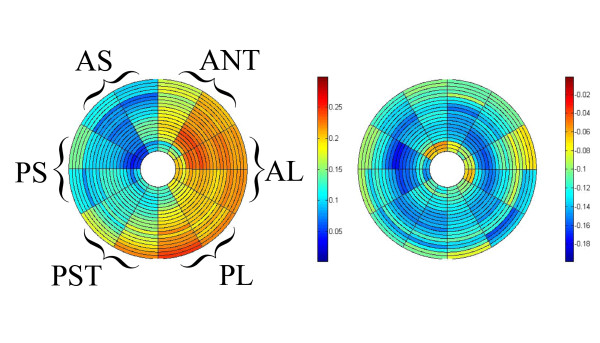
**Bulls-eye plots depict average maximum (A) and minimum (B) principal strains (*ε*_1_, *ε*_3_) for the five animals**. The LV was represented by twenty-two concentric circles starting from the apex (inner circle) to the base (outer circle) and divided into twelve sectors in the circumferential direction. The circumferential sections represent six anatomical regions: anterior septum (AS), posterior septum (PS), posterior wall (PST), posterolateral (PL), anterolateral (AL), and anterior (ANT).

Average maximum and minimum principal strains (***ε***_1_, ***ε***_3_) for the five sheep are summarized in Table 2 and are illustrated by bull's eye plots (Figure 8). Myocardial thickening occurs primarily in the mid-ventricular and basal regions as indicated by the greater maximum principal strains in these segments, while the lateral wall thickens more than the septum. Shortening (***ε***_3_) demonstrates a similar pattern as indicated by a greater minimum principal strain in the mid and basal regions but with more homogeneous distribution than ***ε***_1_, with no dominant contribution from any circumferential segment. Variation in regional principal strain measurements between the animals is slight as indicated by the modest standard deviation of the mean (Table 2).

**Table 2:** Regional LV Principal Strains (mean +/- SD) for the reconstructed ovine left ventricles

	AS		PS		PST		PL		AL		ANT	
***ε***_1_	Aver.	STDEV	Aver.	STDEV	Aver.	STDEV	Aver.	STDEV	Aver.	STDEV	Aver.	STDEV
Apical	0.06	0.05	0.06	0.04	0.1	0.03	0.11	0.08	0.15	0.02	0.1	0.05
Mid	0.11	0.07	0.11	0.03	0.15	0.03	0.19	0.05	0.22	0.03	0.22	0.04
Basal	0.08	0.02	0.1	0.02	0.15	0.03	0.18	0.03	0.19	0.04	0.21	0.05
***ε***_3_												
Apical	-0.04	0.01	-0.12	0.03	-0.08	0.03	-0.08	0.03	-0.08	0.06	-0.05	0.01
Mid	-0.15	0.05	-0.18	0.07	-0.14	0.04	-0.14	0.05	-0.16	0.03	-0.13	0.03
Basal	-0.12	0.03	-0.08	0.04	-0.11	0.05	-0.12	0.05	-0.12	0.02	-0.13	0.03

STDEV, standard deviation of the mean

AS, anteroseptal; PS, posteroseptal, PST, posterior; PL, posterolateral; AL, anterolateral; ANT, anterior

## Discussion

A novel imaging/analysis method was developed that combines a true 3D tagging sequence with 3D OFM motion estimation to accurately track myocardial deformation with high spatial resolution. The addition of a third tag plane to the conventional two dimensional tags enabled through plane motion tracking by providing *z*-plane landmarks and decreasing the ambiguity in this direction. Comparing the manually tracked and measured displacement *in vivo *validated the method. The performance of 3D OFM was further confirmed in the phantom study where displacement was known precisely, indicating accurate estimation. Use of this method to study normal LV function showed qualitative and quantitative functional maps of regional cardiac mechanics, including thickening/lengthening, compression/shortening and twisting. This will help us better understanding normal and pathologic cardiac function, developing effective ways to manage impaired functions, and designing, planning, or evaluating the results of any locally applied therapeutic interventions.

### Optical Flow Methods

Numerous groups have investigated optical flow as a technique to track cardiac motion from tagged images. The 3D OFM applied in this paper was extended from 2D OFM that was previously published by pre-processing the images with a Laplacian filter which reduces the sensitivity to variable brightness [25]. In conventional optical flow methods intensity constancy is assumed [33], which is not valid for tagged CMR due to the relaxation of the magnetization of the spins during the cardiac cycle [25]. To account for the decay of tag pattern, Prince *et al*. [34] modelled the variable brightness by approximating MR parameters. This method is dependent on previous estimation of motion, and its performance degrades as errors accumulate in the MR parameter map. Gupta *et al*. [35] proposed another algorithm to compensate for the time-variable brightness with a linear transformation. This algorithm requires prior knowledge of *T*_1 _and absence of multiplier field motion during the cardiac cycle. The method presented here requires no prior knowledge of relaxation or displacement. Within each estimate stage, the image is decimated multiple times to create a pyramid of coarse-to-fine resolution levels. At each pyramid level multiple iterative estimates of motion are computed and compared to the reference image using SSD as a measure of fit, thus providing faster computation in displacement estimation and eliminating the inaccuracy inherent in the prior methods.

Previous 2D studies have shown that tag pattern frequency influences the accuracy of optical flow in tracking cardiac motion [34]. These results support our conclusion that tag frequency improves accuracy as demonstrated by the increased in-plane tracking correlation with the inclusion of a third tag plane. However, accuracy does decrease above a maximum tag frequency due to the inability to distinguish adjacent tag lines. Also, several additional factors must be taken into account when choosing the appropriate tagging protocol, such as pixel size, SNR, tag thickness, and the magnitude of tissue displacement. Furthermore, the inclusion of an oblique third plane decreases the ambiguity in through-plane pixel tracking for the same rationale as tag frequency for in-plane tracking.

Conventional tagged image analysis tracks the intersection points of the stripes to calculate regional wall motion, which requires that the grid spacing should be smaller than half the heart wall thickness. Additionally, the thickness must be larger than a pixel in order for the stripes to be well resolved [36]. Adult subjects have an average wall thickness of 10-15 mm [37], and best results are obtained using stripes with a thickness 1.5-2 mm separated by about 6-7 mm [8,19,38]. These requirements, however, are relatively difficult to meet in some LV pathologies, such as eccentric dilated hypertrophy and thinned ischemic myocardium post-infarct. As a consequence of limited tags across the myocardium, radial strain has shown the greatest standard error in other reports [39]. In contrast, our method results in accurate radial strain estimation (Table 1). The application of the third tag planes also increases in-plane landmarks, consequently increasing the number of the pixels with high tracking confidence. Tag tracking accuracy has also been shown to be inversely proportional to the tag line contrast-noise ratio (CNR) [40], which decreases due to dephasing and motion and ultimately results in blurring at interfaces such as between the myocardium and cavity. This effect was simulated by addition of Gaussian noise in the phantom study, demonstrated by decreased tag contrast and blurred edges of the tags over the entire image. Decreasing CNR by increasing the relative noise increases the deviation from the expected flow fields, which is more evident at end-systole. RMS error in longitudinal displacement estimation shows more sensitivity to noise, which can be attributed to lower spatial resolution and fewer landmarks in this direction.

The 3D-OFM used in the phantom study has validated its usefulness as a tool for myocardial motion estimation. The acquisition of *in vivo *3D tagged image sets with subsequent 3D OFM allows the tracking of in-plane and through-plane motion. Furthermore, the use of Laplacian filtering on two sequential images followed by displacement vector integration greatly reduces the effect of tag fading on tracking error.

### Left Ventricular Strain in Ovine Models

This study has demonstrated a novel method to image and measure the heterogeneity of the 3D LV wall displacement and deformation in normal animal models without lengthy post-processing. This method provides an alternative solution to study regional LV motion and deformation in normal and diseased hearts.

Studies performed by other groups have investigated the regional displacements and strains generated in the LV during systole. Young *et al*. [41] compared the 3D interpolated displacement and deformation reconstructed from the LV during systole in patients with hypertropic cardiomyopathy (HCM) and normal volunteers using 3D FE model. Xia *et al*. [42] and Park *et al*. [43] also reported a similar study utilizing volumetric models whose parameters are functions in conjunction with physically based deformable modelling frame work. Consistent with findings reported in our study, Young *et al*. reported that in the control group, the apex remained approximately stationary, whereas the base descended toward the apex. Also, the posterolateral wall displaced more than the anteroseptal wall in the longitudinal direction, which might be explained by the spiral motion of LV during systole. Such longitudinal motion patterns have been documented elsewhere as well [39]. However, compared with Young's study less longitudinal displacement at the apex (-1.3 ± 0.8 mm vs. -1.78 ± 1.5 mm) and base (-3.5 ± 1.2 mm vs. -12.6 ± 2.1 mm) were reported, which can be explained by the imaging of an anesthetized animal in this study versus an awake human subject in theirs. In addition, Young *et al*. presented greater in-plane displacement with very little regional variance compared with this study. Moore *et al*. [39] derived the 3D displacement field from tagged breath-held MR images of 31 healthy volunteers, and computed indices of shortening and lengthening based on multiple strain components. This report also presented significant spatial variation in both in-plane and through-plane displacement, with the greatest radial inward displacement observed on the posterior free wall, and the least at the apical-anterior wall, findings which are consistent with those reported in this study.

The presented results show that the maximum principal strain ***ε***_1 _is aligned with the in-plane radial direction. Numerous studies with low spatial density in this direction (usually 2 to 3 tag lines cross heart wall) produced results with higher deviation of ***ε***_1_, which is less reproducible than other parameters. In addition, Moore *et al*. [39] performed displacement field fitting in the radial direction using a first order function, resulting in very little spatial variation in ***E***_*rr *_or ***ε***_1_. However, combination of the 3D high-resolution tagged imaging and analysis presented in this study demonstrated decreased wall thickening from the epicardial towards the endocardial surface, from the base to apex and from the posterior-lateral wall to septum. Similar patterns of ***ε***_1 _are observed from results reported by Young *et al*. [41], and other studies using implanted beads [44], which have higher transmural resolution.

In mid-ventricular to apical regions of the LV, minimum principal strain ***ε***_3 _was aligned within the sub-epicardial surface and angled counter clock-wise from apex to base (viewing from apex), while basal ***ε***_3 _was mostly aligned within the in-plane circumferential direction. The in-plane component of ***ε***_3 _shows higher variance in the longitudinal direction and was greatest in the mid-ventricular region. Myocardial shortening as represented by ***ε***_3 _is more uniform in the circumferential direction. Another finding from this study was that the mid-ventricular section shows the maximum ***ε***_3_, while the majority of this shortening is in the longitudinal direction. In contrast, the basal region shows more circumferential rather than longitudinal shortening. However, due to the difficulties in apical epicardial contouring, the apex was in general defined as a more basal slice in other studies and did not demonstrate the regional strain pattern that was observed here.

Observed inconsistencies of motion/deformation between this study and previous investigations may come from many sources. Dissimilar subject group and modelling methods all contribute to discrepancy. In addition, model-based motion detection methods require the fitting of endocardial and epicardial borders to reconstruct the model geometry [41]. Such approximation procedures may cause errors especially in the apical endocardium, where blood/muscle contrast is insufficient to define the actual borders. Similar difficulties in contouring the myocardial borders also occur where the papillary muscles move in and out of plane and when tag resolution diminishes.

### Limitations and Future Work

A potential limitation of the synthetic phantom was the use of a linear variation in longitudinal displacement, which is not an accurate depiction of normal LV motion. However, this simplification did not diminish the validity of the phantom study to optimize the OFM and tag parameters; since the goal of the phantom was to assess the ability of 3D OFM to precisely recover the known motion applied. Also, the effect of cardiac and respiratory motion was not addressed in the simulation.

An area for potential future study is the use of this method to investigate diastolic deformation. The high spatial resolution and rapid 3D tag acquisition lends itself well to examining the untwisting and relaxation observed during diastole. Also, the high-spatial resolution makes this method applicable to studying the right ventricle due to its irregular shape and thin myocardial wall. In addition, the sub-pixel tracking makes it feasible to relate the 3D motion/deformation to the underlying myocardial fibre structure, which could be used to study the alteration of the extracellular matrix with LV remodelling.

## Conclusions

A novel imaging/analysis method was developed that combines a true 3D tagging sequence with 3D OFM motion estimation to accurately track myocardial deformation with high spatial resolution.

## Competing interests

The authors declare that they have no competing interests.

## Authors' contributions

XC developed the 3D strain algorithm, 3D reconstruction and visualization of the strain field, performed the analysis, and drafted the manuscript. JJP performed image acquisition, and helped draft the manuscript. GI incorporated the 3D OFM algorithm into ImageJ and designed the graphic-user interface (GUI) of the program. ASB performed the animal surgeries. JHG III and RCG helped and participated in study design, manuscript edition, and provided insightful discussions. ZL participated in the analysis, and helped with figures. LD conceived of the study, participated in study design and coordination, and helped draft the manuscript. All authors read and approved the final manuscript.
